# Pigment-Based Chemotaxonomy - A Quick Alternative to Determine Algal Assemblages in Large Shallow Eutrophic Lake?

**DOI:** 10.1371/journal.pone.0122526

**Published:** 2015-03-24

**Authors:** Marju Tamm, René Freiberg, Ilmar Tõnno, Peeter Nõges, Tiina Nõges

**Affiliations:** Centre for Limnology, Institute of Agricultural and Environmental Sciences, Estonian University of Life Sciences, Tartu County, Estonia; CSIR- National institute of oceanography, INDIA

## Abstract

Pigment-based chemotaxonomy and CHEMTAX software have proven to be a valuable phytoplankton monitoring tool in marine environments, but are yet underdeveloped to determine algal assemblages in freshwater ecosystems. The main objectives of this study were (1) to compare the results of direct microscopy and CHEMTAX in describing phytoplankton community composition dynamics in a large, shallow and eutrophic lake; (2) to analyze the efficiency of the pigment-based method to detect changes in phytoplankton seasonal dynamics and during rapid bloom periods; (3) to assess the suitability of specific marker pigments and available marker pigment:chlorophyll *a* ratios to follow seasonal changes in eutrophic freshwater environment. A 5-year (2009-2013) parallel phytoplankton assessment by direct microscopy and by CHEMTAX was conducted using published marker pigment:chlorophyll a ratios. Despite displaying some differences from microscopy results, the pigment-based method successfully described the overall pattern of phytoplankton community dynamics during seasonal cycle in a eutrophic lake. Good agreement between the methods was achieved for most phytoplankton groups - cyanobacteria, chlorophytes, diatoms and cryptophytes. The agreement was poor in case of chrysophytes and dinoflagellates. Our study shows clearly that published marker pigment:chlorophyll a ratios can be used to describe algal class abundances, but they need to be calibrated for specific freshwater environment. Broader use of this method would enable to expand monitoring networks and increase measurement frequencies of freshwater ecosystems to meet the goals of the Water Framework Directive.

## Introduction

Quantification of phytoplankton biomass by taxonomic groups serves as an excellent tool for evaluating water quality and status of aquatic ecosystems [1, 2]. Being a key element of aquatic food webs, phytoplankton has a considerable role in ecological monitoring. European Union has implemented the Water Framework Directive (WFD, [3]), which requires member states to assess water quality in all water-bodies spanning from ground water to coastal marine waters. Phytoplankton is one of the four biological water quality elements used in the WFD to determine the ecological status of a water-body. While boosting the sensitivity of assessment systems, the application of biological indicators brings about new challenges, such as the growing need for time-consuming taxonomic analyses in the expanding monitoring networks and high variability of biological indicators implying increased measurement frequency.

Phytoplankton community composition and abundance are traditionally determined by microscopy and therefore require much time and highly skilful professionals [4]. Despite strong efforts, results may vary notably among specialists and fail to reflect the physiological status of the taxa. Gained very detailed species level information has in general needlessly high resolution for ecological evaluation during monitoring (e.g. [5, 6]). Another major shortcoming is the high lower size limit (5 μm) of standard microscopy that does not allow determining the amount of autotrophic picoplankton (<3 μm) present [7, 8]. The contribution of autotrophic picoplankton to total primary production may reach 50–90% in oligotrophic lakes and oceans [9] and 30–70% in meso/eutrophic lakes [10].

An advantageous alternative to microscopy is the use of phytoplankton marker pigments for quantification of phytoplankton groups. Estimation of phytoplankton composition is usually achieved combining high performance liquid chromatography (HPLC) and a matrix factorization program called CHEMTAX [11]. Unlike morphological, optical, genetic or biochemical methods, the pigment-based method is suitable for regular monitoring as well as ecological studies since: 1) the time needed for analysis is relatively short; 2) much of the work can be automatized; 3) autotrophic picoplankton is included in the analysis [11–13]. A shortcoming of the pigment-based assessment method is that it does not provide high taxonomic resolution beyond the class level. Therefore it has been suggested to use chemotaxonomy together with quick microscopic screening to gain more specific information about dominant species [14–16].

Despite the fact that chemotaxonomy is well acknowledged and wide-spread in oceanographic studies [8, 17–21], it is still moderately used in freshwater systems [15–16, 22–24]. One of the main difficulties relies upon the fact that CHEMTAX requires specific marker pigment:chlorophyll a (Chl a) ratios to calculate taxonomic composition of algae [11]. It has been shown that these ratios vary for each phytoplankton group depending on the environmental conditions such as light and nutrients and therefore are not constant in time and space [9, 16, 24–26].

There is a relatively large variety of marker pigment:Chl a ratios available for marine ecosystems, but very few for different freshwater ecosystems [16, 24, 27]. To gain accurate assays of phytoplankton composition, more precise ratios are needed [11, 28]. In some studies marine marker pigment:Chl a ratios have effectively been used to describe freshwater phytoplankton (e.g [23]). Still most studies agree that more knowledge about pigment ratios in different types of freshwater systems is needed to improve the CHEMTAX estimations and to use it efficiently in regular monitoring. Although recent studies show significant relationships between CHEMTAX and microscopic counts in different aquatic systems [29–31], validation of marker pigment:Chl a ratios with microscopy data is yet needed [24, 25].

The objectives of this study were (1) to compare the results of direct microscopy and CHEMTAX in describing phytoplankton community composition dynamics in a large, shallow and eutrophic lake; (2) to analyze the effectiveness of pigment-based method to detect changes in phytoplankton seasonal dynamics and during rapid bloom periods; (3) to assess the suitability of available specific marker pigments and published pigment:Chl a ratios for following seasonal changes in a eutrophic freshwater environment. To achieve these objectives, a 5-year (2009–2013) parallel phytoplankton assessment by direct microscopy and by CHEMTAX was conducted with a monthly interval in Lake Võrtsjärv, Estonia.

## Materials and Methods

### Study site

**Fig 1:**
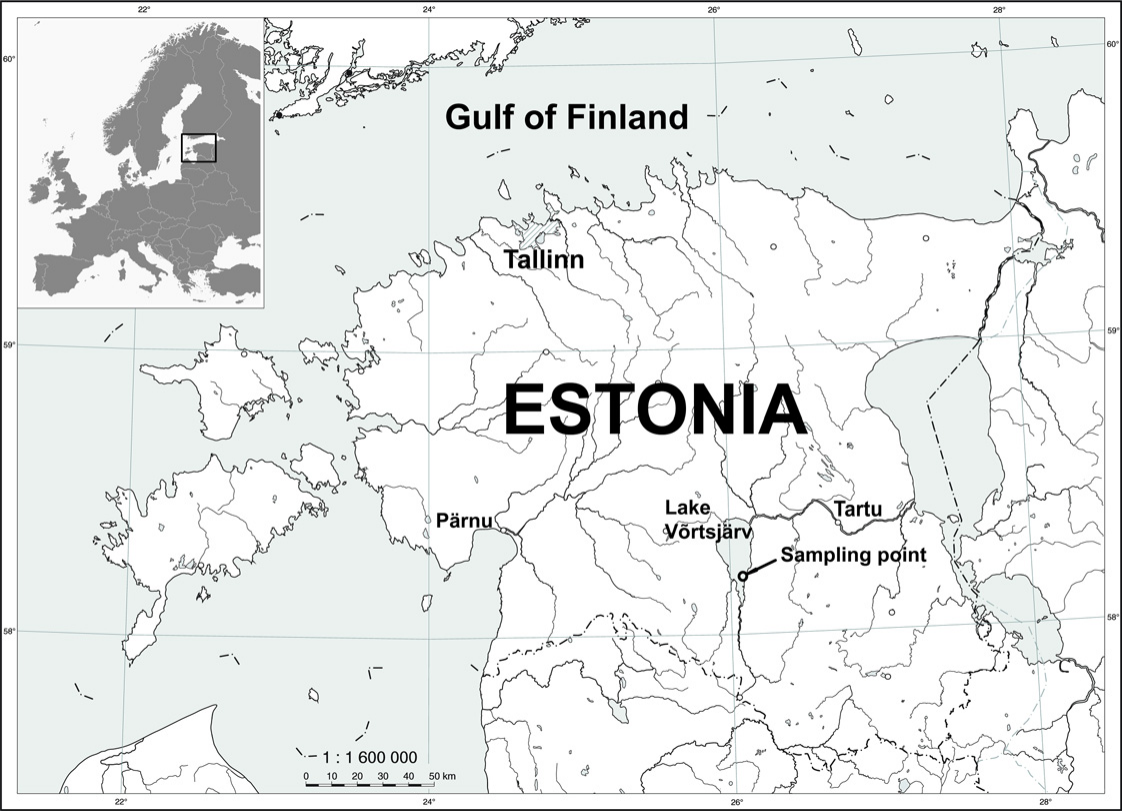
Sampling location in Lake Võrtsjärv (58°12’40”N, 26°06’20”E).

Samples were collected from the large (270 km^2^) shallow (mean depth of 2.8 m, maximum 6 m) and polymictic Lake Võrtsjärv (58°17’N, 26°03’E) located in a shallow preglacial basin in the southern part of Estonia (Fig. 1). Võrtsjärv is eutrophic with an average total phosphorus concentration of 54 μg/l, total nitrogen concentration of 1.6 mg/l and Chl a concentration of 24 μg/l [32, 33]. Cyanobacterial community forms more than 2/3 of the total phytoplankton biomass and is dominated by two slowly growing shade tolerant species *Limnothrix planktonica* (Wolosz.) Meffert and *L*. *redekei* (van Goor) Meffert that persistently build up their biomass over the annual cycle reaching the maximum shortly before ice formation [33, 34]. Other common cyanobacteria species in Võrtsjärv are *Planktolyngbya limnetica* (Lemm.) Kom.-Legn. and *Aphanizomenon skujae* Kom.-Legn. and Cronb. The second largest group consists of diatoms and is mostly dominated by centric diatoms from genera *Aulacoseira* and *Cyclotella* [33]. Ice covers the lake for more than 4 months of the year, on average 135 days.

### Sampling

Depth-integrated samples were collected monthly in 2009–2012 near the lake's deepest point (58°12’40”N, 26°06’20”E, Fig. 1). During the vegetation period of 2013, we used fortnightly sampling frequency to better record short-term changes in phytoplankton community. Chl a was measured spectrophotometrically by HITACHI U-3010 according to Jeffrey and Humphrey [35]. Secchi depth, water temperature and general meteorological conditions were also recorded. Samples were collected with a 4-L Kemmerer sampler with 0.5 m increments starting from approximately 0.5 m below water surface and finishing half meter above sediment surface. These samples were mixed in a 30-L barrel and processed within 2 hours after collection. Subsamples for water chemistry, spectrophotometric measurements, HPLC and microscopy were taken from the depth-integrated water sample in laboratory. No specific permissions were required for any part of the study and field studies did not involve endangered or protected species.

### Microscopic counts

Samples were fixed with 2-% Lugol's iodine solution and stored in cool and dark. Phytoplankton was identified and approximately 400 counting units (cells, colonies, coenobia or trichomes) were counted for each sample under inverted microscope [4]. A transect counting method was used at a 600x magnification. At least one full transect reaching from one edge of the counting chamber to the other along the chamber’s diameter was counted and the full chamber area was checked for less abundant large specimen or colonies. Volume for each counting unit was calculated by applying the formulae of the closest geometric shape [36, 37]. Volume was converted into wet biomass assuming a specific gravity of 1 g/cm^3^. For purposes of this study, microscopic counts of phytoplankton were summed up by main classes: diatoms, cryptomonads, cyanobacteria, chlorophytes, dinoflagellates and chrysophytes.

### HPLC measurements

For pigment analysis integrated lake water samples of 50–200 mL were filtered through Whatman GF/F glass fibre filters (Whatman International Ltd., Maidstone, UK) under gentle vacuum (max. 0.2 bar). Filters were placed in 24-mL plastic tubes and frozen immediately. Samples were stored for not more than 12 months at -70 C° until further analysis. Photosynthetic pigments were extracted from filters with 2 mL mixture of acetone/internal standard and sonicated for 5 minutes with Branson 1210 and kept at -20 C° in darkness for 24 hours. Extracts were filtered through 0.45 μm syringe filters (Millex LCR, Millipore) and stored in darkness at -20 C° for few hours until HPLC analysis.

Reversed-phase high-performance liquid chromatography (HPLC) was applied, using a Shimadzu Prominence (Japan) series system with a photodiode-array (PDA) detector to separate the phytoplankton pigments. A fluorescence detector with excitation wavelength set at 440 nm and emission at 660 nm was used to confirm correct identification and low concentrations of Chl a. A fluorescence detector with excitation wavelength set at 440 nm and emission at 660 nm was used to confirm correct identification and low concentrations of Chl a. The method was adapted from Airs et al. [38] and slightly modified. As an ion-pairing reagent 0.5 M ammonium acetate was added in a volume ratio of 2:3 to each sample before the injection. To avoid chemical decomposition of pigments, the autosampler was cooled down to +5°C [39]. The sample injection volume was 100 μL. Separations were performed in a reversed-phase mode by using two Waters Spherisorb ODS2 3 μm columns (150 mm × 4.6 mm I.D.) in-line with a pre-column (10 mm × 5 mm I.D.) containing the same phase. A binary gradient elution method (Table 1) was used with isocratic holds between 0–2 and 30–43 min.

**Table 1:** HPLC elution scheme and solvents used in the separation of phytoplankton pigments.

	Time, min				
	0	2	30	43	50
Solvent A, %	50	50	100	100	50
Solvent B, %	50	50	0	0	50

Solvent A = 80% methanol: 20% 0.5 M ammonium acetate (pH 7.2) (v:v). Solvent B = 80% methanol: 20% acetone (v:v).

**Fig 2:**
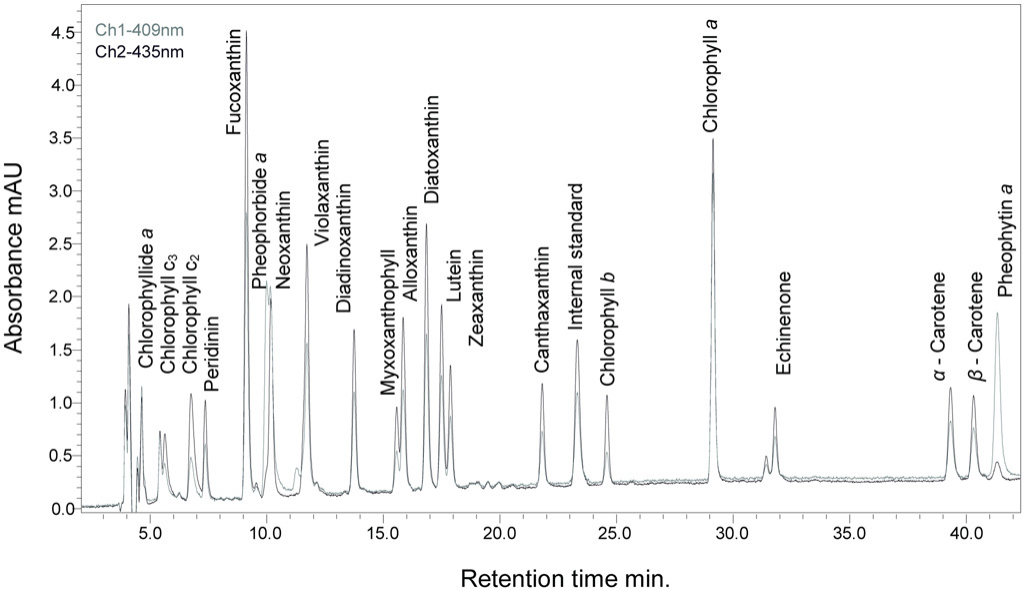
Typical chromatogram of phytoplankton pigment standards. Key pigments, including zeaxanthin and lutein, are well separated.

The flow rate of 0.8 mL/min remained constant during the elution. Absorbance was detected at wavelengths from 350 to 700 nm. The software ‘LC solution ver. 1.22’ (Shimadzu) was applied to collect and analyse the data. The integration of peak areas was made at each pigment absorbance maximum and corrected by internal standard. Commercially available external standards from DHI Water and Environment (Denmark) were used for peak identification and quantification. Standard addition method was used to confirm correct peak identification. Pigments were well separated by this method which was further used for CHEMTAX analysis Fig. 2.

### CHEMTAX analysis

HPLC pigment data was processed with matrix factorization program CHEMTAX (version 1.95) which calculates the relative abundance of phytoplankton classes to total Chl a in a water sample [11]. Published marker pigment:Chl a ratios [24, 40, 41], were applied according to previous knowledge about phytoplankton classes present in Võrtsjärv. Several initial pigment ratio matrices were constructed and tested to find the best marker pigments and marker pigment:Chl a ratios for each phytoplankton group (diatoms, cyanobacteria, chlorophytes, cryptophytes, dinoflagellates and chrysophytes) according to microscopy. Zeaxanthin was set as marker pigment for dominating cyanobacteria group. Fucoxanthin was used for diatoms, lutein and chlorophyll-b for chlorophytes, alloxanthin for cryptophytes, diadinoxanthin and peridinin for dinoflagellates, diatoxanthin and fucoxanthin for chrysophytes. Same initial ratio matrix (Table 2) was used during all five years. Run configuration settings for CHEMTAX were set according to the suggestions of Mackey et al. [42]. Ratio matrix limit was set to 500, initial step size was 10, step ratio 1.3 and cut-off step 1000. To improve biomass estimations, 10 successive runs of CHEMTAX using the output from each run as the input for the next was used as recommended by Latasa [43]. The final ratio matrix is displayed in Table 2.

**Table 2:** Marker pigment:Chl a ratios used for CHEMTAX calculations: a) initial ratio matrix, b) final ratio matrix.

Class/pigment	Diatoxanthin	Fucoxanthin	Alloxanthin	Lutein	Zeaxanthin	Diadinoxanthin	Chlorophyll-b	Peridinin	Chlorophyll-a
(a) Initial ratio matrix									
Chrysophytes	0.025	0.283							1
Dinoflagellates						0.063		0.21	1
Cryptophytes			0.532						1
Chlorophytes				0.148			0.356		1
Cyanobacteria					0.117				1
Diatoms		0.343							1
(b) Final ratio matrix									
Chrysophytes	0.229	0.553							0.218
Dinoflagellates						0.165		0.045	0.791
Cryptophytes			0.261						0.739
Chlorophytes				0.125			0.23		0.645
Cyanobacteria					0.085				0.915
Diatoms		0.186							0.81

### Statistical analysis

Statistical analysis was performed using the statistical package R (version 2.15.3, R Development Core Team, 2013). To assess the relationship between microscopy and CHEMTAX methods, linear regression was used and Spearman rank order correlation coefficient (r_s_) calculated since the data lacked normal distribution.

## Results

**Fig 3:**
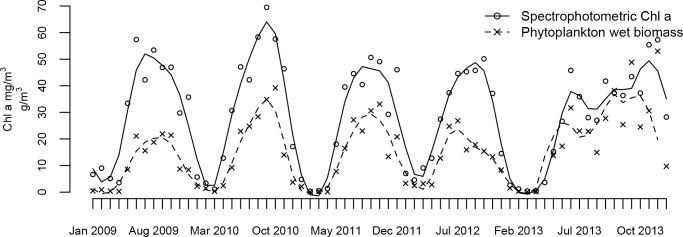
Spectrophotometric chlorophyll-a measurements during years 2009–2013. Loess curve is fit and overlaid on data.

**Fig 4:**
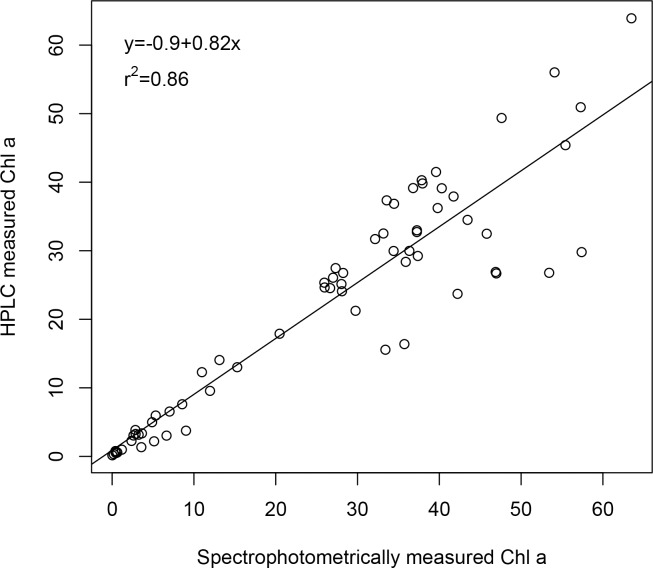
Scatterplot of HPLC measured Chl a and spectrophotometrically measured Chl a in Lake Võrtsjärv (2009–2013).

Spectrophotometrically measured Chl a in Võrtsjärv had a distinct seasonal dynamics with highest values (50–70 mg/m^3^) in summer during cyanobacteria dominance and lowest values (<10 mg/m^3^) in winter (Fig. 3). The maximum Chl a was recorded in 2010 and the minimum (0.3 mg/m^3^) in February 2011. The overall differences between years under investigation were not remarkable. There was a strong positive correlation (r_s_ = 0.93, p<0.001) between spectrophotometrically measured Chl a and HPLC derived Chl a (Fig. 4). Spectrophotometrically measured Chl a tended to overestimate Chl a concentrations for about 15%. Strong correlation (r_s_ = 0.88, p<0.001) was found also between spectrophotometrically measured Chl a and phytoplankton wet biomass. During the vegetation period, Chl a concentration tended to increase approximately 40% more than the biomass. The average Chl a/biomass ratio was 2.3 in January and 1.5 in July.

**Fig 5:**
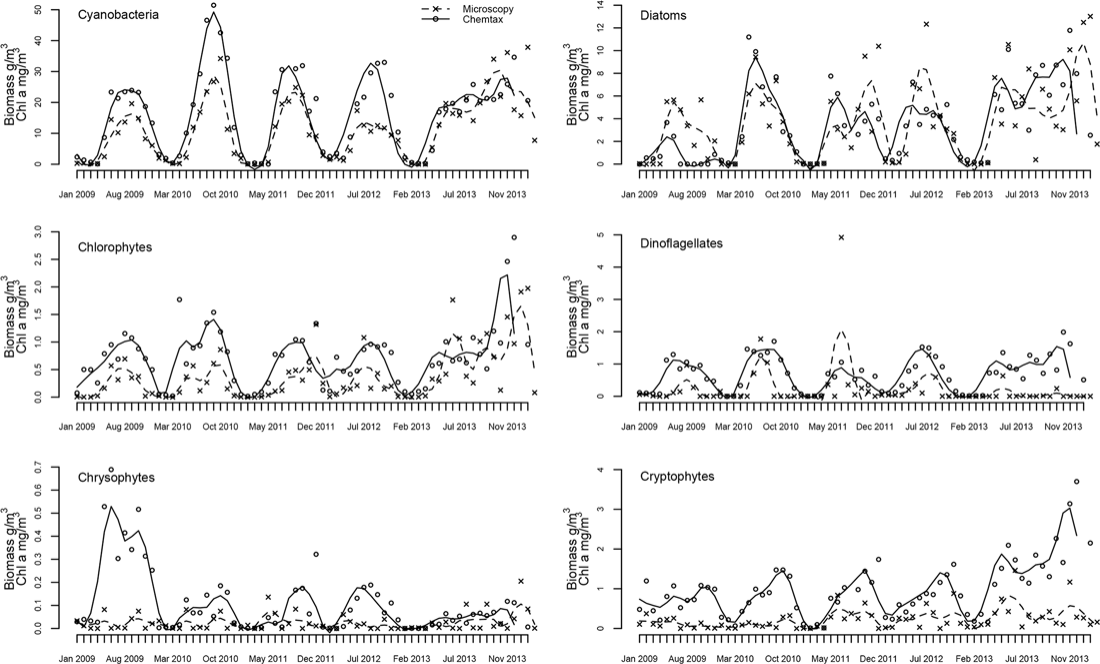
Dynamics of phytoplankton groups in Lake Võrtsjärv (2009–2013) described by microscopy (phytoplankton wet biomass g/m3) and CHEMTAX (Chl a mg/m3). Loess curve is fit and overlaid on data.

Among marker pigments zeaxanthin had the highest concentrations and its abundance patterns followed Chl a dynamics (Figs. 3 and 5). Similarly to Chl a, zeaxanthin values peaked in summer 2010 with the maximum value reaching 4.57 mg/m^3^.

### Differences between microscopy and CHEMTAX

**Fig 6:**
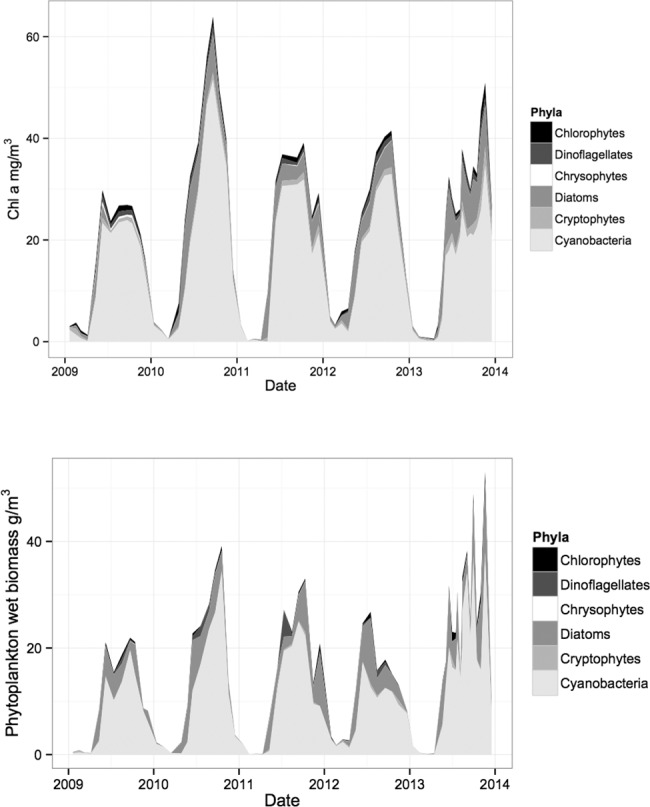
Dynamics of phytoplankton communities in Lake Võrtsjärv (2009–2013) according to CHEMTAX (upper) and microscopy (lower).

**Fig 7:**
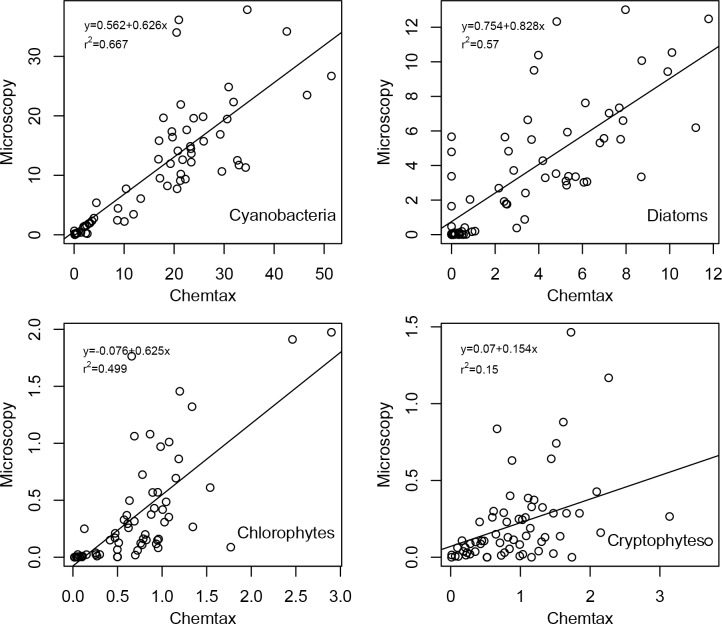
Linear regression of CHEMTAX and microscopy results for cyanobacteria, diatoms, chlorophytes and cryptophytes in Lake Võrtsjärv (2009–2013).

Overall both techniques had similar outcomes concerning studied phytoplankton class abundances (Fig. 6). Dominant groups (cyanobacteria and diatoms) were detected and well separated from other phytoplankton groups which had minor contribution to total phytoplankton biomass. Best correspondence between the two assessment methods was achieved for cyanobacteria—both methods reflected the summer maxima, winter minima and had similar relative peak sizes (Fig. 5). The scatterplot of microscopy and CHEMTAX results (Fig. 7) also suggests a very good agreement between the methods. Zeaxanthin containing cyanobacteria accounted for about 73% of total Chl a during vegetation periods. Microscopy revealed that the community was nearly always dominated by *Limnothrix planktonica*, on rarer occasions by *L*. *redekei*.

Seasonal changes were well traced for most groups by both methods though there were inconsistencies for some classes (e.g. diatoms, chrysophytes). Similarly to microscopy, CHEMTAX showed diatoms being the second largest group in phytoplankton community (mostly dominated by genera *Aulacoseira* or *Synedra*), but the relative share of the group varied considerably in different years depending on the assessment method (Fig. 5). E.g., CHEMTAX calculations suggested significantly (p<0.01) lower amount of diatoms in 2009 compared to other years while microscopy did not indicate any significant year-to-year differences. The match between CHEMTAX estimations and microscopy for diatoms was weaker than for cyanobacteria being at about the same level as for chlorophytes (Fig. 7). During the study period, chlorophytes showed no definite dominant species.

Another inconsistency between the methods occurred in chrysophytes (Fig. 5) for which CHEMTAX estimated significantly higher abundance (p<0.001) in 2009 whereas microscopy didn't detect any significant differences between the years. The overall diversity of chrysophytes was low and the community was mostly dominated by *Dinobryon* spp. followed by few less abundant species. A similar contradiction between the methods appeared with cryptophytes in year 2013. Alike chrysophytes, the community of cryptophytes was quite homogeneous consisting mostly of *Cryptomonas* spp. and rarely of *Rhodomonas* sp. General agreement between the methods for chrysophytes and cryptophytes was also rather weak (Fig. 5).

Spearman rank order correlations between microscopy and CHEMTAX results were significant for most assessed phytoplankton groups (cyanobacteria, diatoms, chlorophytes, cryptophytes and chrysophytes) except dinoflagellates (Table 3).

**Table 3:** Spearman rank order correlation coefficients of phytoplankton classes assessment by microscopy and CHEMTAX in Lake Võrtsjärv in 2009–2013.

	r_s_	p
Cyanobacteria	0.87	<0.001
Chlorophytes	0.77	<0.001
Diatoms	0.74	<0.001
Cryptophytes	0.52	<0.001
Chrysophytes	0.27	<0.05
Dinoflagellates	0.21	>0.05

## Discussion

This study covers a five year period of parallel chemotaxonomic and microscopic analysis of phytoplankton community of Lake Võrtsjärv. Our findings support the hypothesis that pigment-based chemotaxonomy can successfully describe the phytoplankton community composition in a large, shallow and eutrophic lake. Good correlations between these two methods were obtained for the more abundant phytoplankton groups—cyanobacteria, diatoms and chlorophytes (Table 3).

The study highlights the need for preliminary knowledge about the phytoplankton community of the lake since this enables selecting the right marker pigments to be used in the initial ratio matrix for CHEMTAX calculations [26, 44]. For the current study, pigment ratios were acquired from previously published works which were not exclusively done in eutrophic freshwaters [24, 40, 41]. Different matrixes were tried out before constructing the ultimate input matrix which gave most comparable results with phytoplankton microscopy of Võrtsjärv. Same ratios were applied for all years to analyse their capability of responding to seasonal changes.

In addition, phytoplankton wet biomass and Chl a concentrations (HPLC and Spectrophotometry) were compared. Discrepancy between phytoplankton wet biomass and HPLC derived Chl a was most likely caused by photoacclimation to deteriorating light conditions and changes in taxonomic composition of phytoplankton community over summer. A similar shift has been shown in several other studies [45, 46]. Our finding that spectrophotometry gave 15% higher Chl a concentrations than HPLC was well supported by the study of Sørensen et al. [47] in which 15–20% higher spectrophotometric values were observed during an intercomparison exercise. Such disagreement in Võrtsjärv can occur due to the Chl a degradation products (pheopigments) and accessory pigments with overlapping spectra [48, 49]. The HPLC technique measures only the Chl a that matches the commercial standard, while spectrophotometry includes also other Chl a derivatives such as divinyl Chl a, Chl a allomer, and Chl a isomer. Presence of other pigments (e.g. chlorophyll b, c, and the respective degradation products) is known to considerably interfere chlorophyll-a determination [50].

In the present study we observed significant correlations between microscopy and CHEMTAX for most of the phytoplankton groups. Strong correlation between zeaxanthin-derived and microscopically counted cyanobacteria (r_s_ = 0.87, p<0.001) are in good agreement with Schlüter et al. [24] who also achieved very high correlation (r = 0.95, p<0.05) for cyanobacteria in eutrophic lakes using only zeaxanthin as diagnostic pigment. Since zeaxanthin:Chl a ratios are sensitive to light, it is often suggested that the results would benefit from the use of additional pigments—e.g. myxoxanthophyll or echinenone [24, 51]. While this might be inevitable in some cases, our findings show that it is not always required. While testing some of the initial pigment ratios, better consistency was achieved excluding echinenone as additional marker pigment. Same approach was used by Lauridsen et al. [16] in order to avoid errors due to other echinenone-containing algae with high chlorophyll-b levels (e.g. chlorophytes). Using only zeaxanthin as marker pigment for cyanobacteria may avoid distortions that are caused by pigments of other species in phytoplankton community. The good agreement of the two methods tested in Võrtsjärv is likely related to the fact that phytoplankton community was nearly always dominated by same species. This minimised the potential fluctuations of the pigment composition resulting from the variation of algal species.

The correlation between CHEMTAX and microscopy results was also strong for diatoms (r_s_ = 0.74, p<0.001) and chlorophytes (r_s_ = 0.77, p<001). In eutrophic conditions an even better agreement between the methods (r = 0.89) has been previously reported for diatoms [24] whereas in meso- and oligotrophic freshwaters much weaker relationship was achieved by Laurindsen et al. [16] although the same marker pigment was used. This stresses the importance of applying appropriate marker pigment:Chl a ratios.

In the current study the CHEMTAX vs. microscopy correlation coefficient for chlorophytes was significantly higher than found in several other freshwater studies (e.g. [16, 24, 40]). A comparably strong correlation was achieved by Garibotti et al. [20] in Antarctic coastal waters, although only Chl b was used as a marker pigment. Usually additional pigments such as lutein or violaxanthin are used [15, 24].

The agreement between the two methods was generally poorer for less abundant phytoplankton groups (Table 3) such as cryptophytes, dinoflagellates and chrysophytes. These groups never dominated during the vegetation period and the discrepancies were most likely caused by higher counting errors of these minority groups. Although their biomasses were mostly built up by single genera (by *Cryptomonas* in cryptophytes, *Peridinium* in dinoflagellates, and *Dinobryon* in crysophytes), this small variability of species in certain phytoplankton groups alone could not guarantee good agreement of the different assessment methods.

CHEMTAX generally performs better when the number of marker pigments per class is small or when the cellular content of one of the marker pigments is relatively high as peridinin in dinoflagellates [25]. To the contrary, in our study we did not find significant correspondence of the two methods for dinoflagellates. In our study the CHEMTAX peaks for dinoflagellates tended to be much broader than the microscopy biomass peaks. Since peridinin is an unambiguous marker for dinoflagellates, this refers to the fact that dinoflagellates are present in the phytoplankton community, but are underestimated by microscopy. The biomass of dinoflagellates in Võrtsjärv is very small but is mostly built up by large individuals that both increase the variability of counting results. Moreover, the poor light conditions during summer due to supended particles and high biomass of other phytoplankton species could increase the production of peridinin in light harvesting complexes to sustain the same amount of biomass. This could also add some disagreement to the results achieved by the two methods as seen in Fig. 5.

Cryptophytes were strongly overestimated (especially in 2013) by CHEMTAX (Fig. 5) while good agreement between the methods using same marker pigment and similar marker pigment:Chl a ratios have been achieved by other authors [16, 24]. On one hand, this could imply to specific species composition of phytoplankton community in Võrtsjärv that might cause the difference in alloxanthin:Chl a ratio compared to other lakes. The pigments involved in photoprotection (such as alloxanthin) may increase considerably with high irradiance levels independently from Chl a and can hence cause significant differences in marker pigment:Chl a ratios [9], [24], [26]. Schlüter et al. [24] showed large variability of alloxanthin:Chl a ratios of cryptophyte cultures under different light conditions. This may be another factor causing disagreement between the two methods compared in current study.

We found very weak correlation between CHEMTAX and microscopy results for chrysophytes (Fig. 5, Table 3). Laurindsen et al. [16] also found no significant correspondence for chrysophytes in meso/oligotrophic conditions while, on the contrary, Schlüter et al. [24] had excellent agreement between the two methods for chrysophytes in eutrophic conditions. The weak correlation in our study could be caused by low counting precision of this minority group but also by errors in quantification of minor pigments (such as diatoxanthin in case of chrysophytes) which are shared by several phytoplankton classes and thus may contribute to the discrepancies [22]. Diatoxanthin can also interconvert with diadinoxanthin as was shown in Freiberg et al. [52] that makes the pigment-based chemotaxonomic assessment of chrysophytes and dinoflagellates more difficult. Still it is likely that a separate intercalibration exercise based on high precision counts of the minority groups might yield more consistent results and that the high sensitivity of the CHEMTAX method could prove one of its main advantages lowering the uncertainty of the minority group biomass estimates.

A shortcoming of this study is the fact that we have no microscopy data on picoplankton of Lake Võrtsjärv for the years 2009–2013. Phytoplankton was assessed by both microscopy and CHEMTAX, but picoplankton was included only in the latter. That could account for a considerable amount of variation between the two methods, especially in case of cyanophytes. In future studies, picoplankton counts should always be included.

It is obvious that using pigment-based chemotaxonomy saves time and money. Broader use of this method would enable to expand monitoring networks and increase measurement frequencies as well as precision, especially for the minority groups. Our study confirmed the suggestion by Latasa [43] that generic input of pigment ratio matrixes combined with successive runs of CHEMTAX can give good enough biomass estimates for regular monitoring. Adding to it a quick microscopic screening of dominant taxa would meet the needs of the WFD [8, 15]. Further studies are needed to determine more precise marker pigment:Chl a ratios in different freshwater environments to improve CHEMTAX estimations and define the main sources of errors.
